# Recent Updates in Venetoclax Combination Therapies in Pediatric Hematological Malignancies

**DOI:** 10.3390/ijms242316708

**Published:** 2023-11-24

**Authors:** Maria Leśniak, Justyna Lipniarska, Patrycja Majka, Monika Lejman, Joanna Zawitkowska

**Affiliations:** 1 Student Scientific Society of Department of Pediatric Hematology, Oncology and Transplantology, Medical University of Lublin, 20-093 Lublin, Poland; marialesniak68@gmail.com (M.L.); justyna.lipniarska@gmail.com (J.L.); patrycja_majka@wp.pl (P.M.); 2 Independent Laboratory of Genetic Diagnostics, Medical University of Lublin, 20-093 Lublin, Poland; monika.lejman@umlub.pl; 3 Department of Pediatric Hematology, Oncology and Transplantology, Medical University of Lublin, 20-093 Lublin, Poland

**Keywords:** venetoclax, Bcl-2 inhibitors, pediatric hematology, leukemia, treatment

## Abstract

Venetoclax is a strongly effective B-cell lymphoma-2 inhibitor (BCL-2) with an ability to selectively restore the apoptotic potential of cancerous cells. It has been proven that in combination with immunotherapy, targeted therapies, and lower-intensity therapies such as hypomethylating agents (HMAs) or low-dose cytarabine (LDAC), the drug can improve overall outcomes for adult patients with acute myeloid leukemia (AML), chronic lymphocytic leukemia (CLL), and multiple myeloma (MM), amongst other hematological malignancies, but its benefit in pediatric hematology remains unclear. With a number of preclinical and clinical trials emerging, the newest findings suggest that in many cases of younger patients, venetoclax combination treatment can be well-tolerated, with a safety profile similar to that in adults, despite often leading to severe infections. Studies aim to determine the activity of BCL-2 inhibitor in the treatment of both primary and refractory acute leukemias in combination with standard and high-dose chemotherapy. Although more research is required to identify the optimal venetoclax-based regimen for the pediatric population and its long-term effects on patients’ outcomes, it can become a potential therapeutic agent for pediatric oncology.

## 1. Introduction

### 1.1. Apoptosis

Apoptosis is a major form of programmed cell death, which is very important for the development and functioning of multi-cellular organisms. It regulates cell number, the removal of structures, and tissue sculpting and also protects against pathogens [1]. Apoptosis deregulation can lead to many diseases—cancer, autoimmune diseases, viral infections, or neurodegenerative disorders [2].

Caspases play a key role in the mechanism of apoptosis as they are both the initiators (caspase-8, -9, and -10) and executioners (caspase-3, -6, and -7) of the process. There are two main pathways by which caspases can be activated—the intrinsic (mitochondrial) pathway, initiated by microenvironmental disturbances, and the extrinsic (death receptor) pathway, activated by disturbances of the extracellular microenvironment [3]. The intrinsic pathway is initiated by, among others, genetic damage, oxidative stress, endoplasmic reticulum stress, hypoxia, or mitotic defects. This pathway is the result of increased mitochondrial penetrability and the release of proapoptotic molecules that normally reside in the mitochondrial intermembrane space into the cytoplasm [4].

### 1.2. B-Cell Lymphoma 2 (Bcl-2) Protein Family

The B-cell lymphoma 2 (Bcl-2) protein family regulates mitochondrial apoptosis. The members of the Bcl-2 family are classified into three subgroups, depending on the composition of typical BH (Bcl-2 Homology) domains, listed from BH1 to BH4, and their involvement in apoptosis regulation [5]. The BH1 and BH2 domains of Bcl-2 are needed for dimerization with proapoptotic proteins. The BH3 domain is of primary importance for the interaction between proapoptotic and antiapoptotic proteins and is present in all family members. The amino-terminal BH4 domain is mainly found in the antiapoptotic Bcl-2 family members [6].

The proteins are categorized into:

- Antiapoptotic, e.g., BCL-2, BCL-XL, BCL-W, and MCL-1;
- BH3-only (proapoptotic), e.g., BIM, BID, PUMA, NOXA, BIK, and BAD;
- Pore-forming or ‘executioner’ (proapoptotic), e.g., BAX, BAK, and BOK.

Subfamily categorization is based on the BH and transmembrane domains, anti- or proapoptotic function status, and pore-forming ability [7,8].

**Figure 1:**
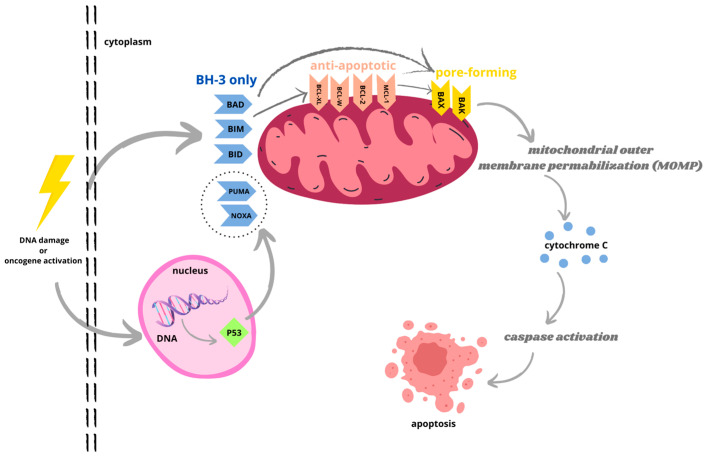
Bcl-2 protein family regulates mitochondrial apoptosis via intrinsic pathway initiated by toxic factors, genetic damage, or oncogene activation. The interaction between antiapoptotic and pro apoptotic Bcl-2 proteins prevents cell death. Activated BH3-only proteins bind to antiapoptotic proteins, located in outer mitochondrial membrane (OMM), resulting in release of pore-forming effector proteins BAX and BAK, which cycle between OMM and cytosol. Activated BH3-only proteins can also directly activate BAX and BAK. The translocation of BAX and BAK induces mitochondrial outer membrane permeabilization (MOMP) and cytochrome c release, followed by formation of apoptosome and caspase activation. Caspase cascade causes destruction of apoptotic cells.

The translocation of proapoptotic proteins BAX and BAK induces mitochondrial outer membrane permeabilization and cytochrome c release, followed by caspase activation (Figure 1). BH3-only proteins BID and BIM promote mitochondrial permeabilization via the activation of BAX and BAK, while BH3 proteins BAD, BIK, and PUMA bind and oppose the activation of antiapoptotic proteins. In contrast, antiapoptotic proteins prevent BAX/BAK-dependent mitochondrial outer membrane permeabilization via both the direct inactivation of BAK and BAX and via the sequestration of BH3-only proteins [9,10,11]. The proapoptotic proteins promote the mitochondrial release of cytochrome-c, whereas the antiapoptotic proteins regulate apoptosis by blocking this release. The delicate balance between these two groups determines whether a cell survives or dies [4]. Antiapoptotic proteins are often exploited by tumor cells to avoid death, thus playing an important role in carcinogenesis and in the acquisition of resistance to various therapeutic agents. Therefore, antiapoptotic proteins represent attractive targets in cancer therapy [12].

### 1.3. Bcl-2 Inhibitors

In recent years, work has been carried out to selectively target Bcl-2 proteins by creating BH3 mimetics that bind to hydrophobic grooves of the pro-survival proteins with high affinity. As a result, a number of Bcl-2 inhibitors have been developed [13].

The first highly selective Bcl-2 inhibitor, ABT-737, and its clinical derivative navitoclax (ABT-263) target BCL-2, BCL-W, and BCL-XL. Navitoclax is a BH3 mimetic drug which binds strongly to the BH3 domain of BCL-2 antiapoptotic members. Navitoclax binds to the BH3 binding groove of BCL-2 proteins located in the cytoplasm, causing the displacement of proapoptotic BH3-only protein BIM from BCL-2. The release of BIM causes apoptosis. Navitoclax alone successfully treats small-cell lung cancer and acute lymphocytic leukemia, whereas in combination therapy for solid tumors, it enhances the therapeutic effect of other chemotherapeutic agents [14,15,16].

Another advancement was the discovery of obatoclax (GX15-070). It is a less selective Bcl-2 inhibitor that antagonizes BCL-2, BCL-XL, BCL-W, and MCL-1. Obatoclax is a synthetic indole bipyrrole derivative of bacterial prodiginines. It is a small-molecule inhibitor of the antiapoptotic proteins of the Bcl-2 family [17,18,19]. Several phase I and II clinical trials have shown its only modest efficacy in the treatment of solid tumors and hematological malignancies [20]. Obatoclax may be more effective when used in combination with other anticancer therapeutics [21].

The next innovation was the development of venetoclax (VTX; ABT-199). It is a highly selective oral Bcl-2 inhibitor with high affinity to BCL-2 and lower affinity to BCL-W and BCL-XL—a molecule crucial for platelet survival. VTX shows activity in BCL-2-dependent hematologic malignancies, especially in chronic lymphocytic leukemia (CLL) [13,22]. The maximum plasma concentration is reached 5–8 h post-dose, and the elimination half-life ranges between 17 and 41 h after a single oral dose. Bioavailability is increased by food, and it is primarily metabolized via the CYP3A pathway and through the hepatic/fecal system [23].

This review describes the role of VTX in the modern treatment of pediatric hematologic malignancies, especially AML and ALL. We show the most recent clinical studies using VTX as a therapeutic option in independent and combined treatment. We also discuss emerging VTX resistance and possible therapeutic solutions.

### 1.4. Venetoclax in Adult Hematology

VTX has been successfully granted approval by the U.S. Food and Drug Administration (FDA) as a treatment option for previously untreated patients with CLL or small lymphocytic lymphoma (SLL), with or without 17p deletion, in combination with obinutuzumab [24,25], as well as for newly diagnosed acute myeloid leukemia (AML) patients who are 75 years or older, or who have comorbidities precluding intensive induction chemotherapy in combination with azacitidine, decitabine, or low-dose cytarabine (LDAC) [26] (Table 1).

In relapsed and refractory AML patients, the efficacy and safety of VTX monotherapy were first explored in a phase II study, which showed low efficacy with an overall response rate (ORR) of 19% [27], whereas VTX combination AML therapies with DNA methyltransferase inhibitors (DNMTis) or LDAC demonstrated promising results [28]. In a study of 145 patients over 60 years, VTX in combination with DNMTis was associated with complete remission (CR) or complete remission with incomplete hematologic recovery (CRi) in 67%, with a median overall survival of 17.5 months [29]. In 82 patients over 60 years receiving VTX in combination with LDAC, the CR/CRi rate was 54%, and the median overall survival (OS) was 10.1 months [30]. Despite those promising responses, primary resistance and clonal evolution leading to adaptive resistance remains an important theme in AML. Recent studies have illustrated the complex and polyclonal nature of resistance to targeted therapeutics [31]. *TP53* mutations are especially related to inferior response rates, shorter disease response, and higher minimal residual disease positivity in newly diagnosed AML patients treated with a combination of VTX and decitabine [32].

In view of its beneficial efficacy and tolerable toxicity profile, VTX has become a therapeutic option for the management of de novo and relapsed refractory CLL, demonstrating durable responses regardless of adverse prognostic features such as deletion (del) (17p) [33,34,35]. In a cohort of 158 patients, mainly with relapsed and refractory (RR) CLL with del (17p), treatment with VTX established promising tolerability and durable responses, including an ORR of 77%, undetectable minimal residual disease (uMRD) in peripheral blood (PB) of 30%, and estimated 24-month progression free survival (PFS) of 50% [36]. Retrospective data from 683 patients with CLL, treated with ibrutinib, idelalisib, or VTX after ibrutinib therapy failure, demonstrated better outcomes in those treated with VTX (ORR 79%) versus idelalisib (ORR 46%). Furthermore, in the case of kinase inhibitor (KI) failure, alternate KI or VTX therapy appears superior to chemoimmunotherapy variations [37]. Combination therapy with anti-CD20 monoclonal antibodies (mAbs) and other small molecules in CLL has been the subject of interest in order to achieve deeper and more durable responses and allow for fixed-duration therapy [33,38].

**Table 1:** Venetoclax (Venclexta^®^) FDA approval history.

Date	Approval	References
11 April 2016	For the treatment of patients with CLL with a 17p deletion, as detected with an FDA-approved test, who have received at least one prior therapy	[39]
8 June 2018	In combination with rituximab for the treatment of people with CLL or SLL, with or without a 17p deletion, who have received at least one prior therapy	[24]
21 November 2018	Accelerated approval to venetoclax, in combination with azacitidine or decitabine or low-dose cytarabine for the treatment of newly diagnosed AML in adults who are age 75 years or older, or who have comorbidities that preclude the use of intensive induction chemotherapy	[40]
15 May 2019	In combination with obinutuzumab for previously untreated patients with CLL or SLL	[25]
16 October 2020	Full approval of venetoclax in combination with azacitidine, decitabine, or low-dose cytarabine (LDAC) for the treatment of newly diagnosed AML in adults 75 years or older, or who have comorbidities that preclude the use of intensive induction chemotherapy	[26]

For many patients, especially those with high-risk disease, VTX-based therapy is better tolerated and more effective than traditional chemoimmunotherapy.

Moreover, there is the novel Bcl-2 inhibitory compound lisaftoclax (APG-2575) that is currently undergoing clinical evaluation upon FDA permission [41] and has been granted four Orphan Drug Designations (ODDs) by the FDA for the treatment of patients with AML, CLL, Waldenström macroglobulinemia (WM), and multiple myeloma (MM) [42].

## 2. Pediatric AML

Regardless of significant advances in the treatment of AML and the continuous expansion of new treatment regiments, there is a group of patients who are unqualified or impervious to intensive induction chemotherapy, resulting in a poor prognosis and restricted therapeutic options [43,44]. Children with relapsed or refractory acute myeloid leukemia have poor treatment outcomes and overall survival. Despite relapse prevention being the aim of initial therapy, around 30% of pediatric AML patients will later develop bone marrow relapse, with an overall survival of less than 40% [45]. Since it has been proven that some AML blast cells display high levels of BCL-2 protein, mainly amongst chemotherapy-resistant patients [46,47,48,49], antiapoptotic proteins are seen as a promising therapeutic target. Despite encouraging results being reported for VTX usage both in monotherapy and in combination therapies in clinical studies of adult patients with AML [27,49,50], not much is known about VTX administration and efficacy in younger patients. Due to the fairly low incidence of childhood cancers, conducting a pediatric clinical study faces many limitations including a small sample size, heterogeneous cohort, lack of a control group, short duration of follow-up, and occurrence of disease progression during single-agent studies.

### 2.1. Clinical Studies in Relapsed/Refractory AML

VTX is currently the subject of many ongoing phase I/II clinical trials to estimate the virtue and tolerability of this agent in this population (Table 2). The most notable study to date was carried out by Karol and colleagues. They aimed to ascertain the tolerance of VTX in combination therapy with standard and high-dose chemotherapy in pediatric patients with relapsed/refractory AML or ambiguous lineage leukemia. The conducted phase I dose escalation study (NCT03194932) proposed the safety and activity of BCL-2 inhibitor in combined therapy. A total of 36 patients were given VTX in 28-day cycles at 240 mg/m^2^ or 360 mg/m^2^, in combination with cytarabine, with or without intravenous idarubicin. The recommended phase II dose was established at 360 mg/m^2^. The overall response was observed in 69% of the 35 patients assessed after the first cycle, with a notable 70% complete response rate among the 20 patients treated at the recommended phase II dose. Among the patients, 66% developed febrile neutropenia and 16% developed invasive fungal infections. The findings also highlight the need for such a treatment combination evaluation in newly diagnosed patients with high-risk AML [51]. These encouraging data prompted a study by Place et al. to establish the dose-limiting toxicity, pharmacokinetics, and preliminary efficacy of VTX monotherapy in an open-label, global phase I two-part study (EudraCT 2017–000439–14; NCT03236857) amongst pediatric and young adult patients (AYA) with relapsed/refractory malignancies. During part 1, younger patients with any relapsed/refractory tumor type who had no available curative options were enrolled. Participants were given VTX daily with a two- or three-day dose ramp up to 800 mg, weigh and age-adjusted, over the course of 9 months. After the first assessment, patients may have received VTX in combination with chemotherapy, beginning at week 4 in patients with hematologic malignancies. During part 2, patients with relapsed/refractory acute lymphoblastic leukemia (ALL), AML, non-Hodgkin’s lymphoma (NHL), or neuroblastoma were enrolled in four cohorts; the outcomes are yet to be presented [52].

### 2.2. Other Studies on Venetoclax Combination Therapies in AML 

Apart from those early-phase trials, the available literature discussing VTX in pediatric/AYA patients with hematologic malignancies contains a couple of single-institution reports [53,54,55,56,57,58,59] (Table 3) or multicenter retrospective analyses [60,61,62,63,64,65,66].

Recent studies support the usage of VTX-containing regimens in myeloid malignancies, especially as a linking therapy for allogeneic hematopoietic stem cell transplantation (HSCT) [60,61,62,63]. Niswander et al. analyzed 37 pediatric patients with relapsed/refractory acute leukemias, including many with high-risk cytomolecular genetic features treated with VTX in combination with hypomethylating agent (HMA) with or without CD33 antibody gemtuzumab ozogamicin. The median minimal residual disease (MRD) level was 0.5%, with 14 patients (n = 12 AML) achieving a CR with MRD-negative remission (38% of 37 treated patients). Patients’ responses to the regimen were typically achieved within one cycle of therapy or not at all. Successful remission induction was HSCT-enabling for 11 patients with AML [61]. A multicenter retrospective analysis evaluated VTX with HMA azacitidine or decitabine, or with a combination of cytotoxic agents, such as cytarabine, fludarabine, idarubicin, or doxorubicin, in 31 pediatric patients with high-risk myeloid malignancies who had received previous lines of therapy. The median dose of 350 mg/m^2^ VTX was administered daily within a median of two cycles. The response rate was satisfying, with an overall response rate of 71% and a CR of 51.6%. Twenty patients received allogeneic HSCT at a median time of 3.3 months from the start of treatment and were alive at the end of follow-up (7.7 months) [62]. Moreover, a retrospective report from Children’s Hospital Colorado by Winters et al. on the use of azacitidine (AZA)/VTX combination among six patients with AML revealed that all responders achieved minimal residual disease negativity and three of them proceeded to HSCT [63].

The most common adverse events (AEs) found were a prolonged depletion of all blood cell lines, especially neutropenia, and severe blood, pulmonary, and skin infections, including bacteriemia, which are consistent with other published data on VTX in pediatric patients with acute leukemias [65,66]. The overall response rate was comparable to that seen in heavily pretreated adult patients with AML who received similar VTX combination therapies [50]. Most patients received maximal benefit within one to two cycles of VTX-based therapy, and all durable responses were followed by HSCT, indicating those regimens are likely to become a bridge therapy for allogeneic hematopoietic stem cell transplantation rather than a definitive therapy.

### 2.3. Genetic Sensitivity and Resistance to Venetoclax

With acute myeloid leukemia being a molecularly heterogeneous disorder, genetic lesions have been linked to particular clinical features, therapy response, and patients’ outcomes, leading to improvements in risk stratification. There are favorable (*RUNX1-RUNX1T1*, *CBFB-MYH11*, *NPM1*, and *CEBPA bZIP*) and unfavorable (*MECOM*, *DEK-NUP214*, *KMT2A*, *NUP98*, *FLT3/ITD*, *WT1*, monosomy 7, monosomy 5, and *TP53*) pediatric genetic markers that are being used to guide practitioners through patients’ management [67,68]. Amongst the available data discussing clinical experience with VTX in children, the most recurrent structural rearrangements or sequence variants observed were *KMT2A* rearrangements, *FLT3* alterations, and *NPM1* mutations; many patients who obtained CR had a particular molecular subtype of the malignancy or a cancer predisposition syndrome [51,60,61,62,63,64,65,66]. It is important to establish which molecular subtypes of pediatric malignancies might display specific VTX vulnerability, despite no evident mechanistic links to BH3 mimetic responses. *KMT2A* is a frequently rearranged gene in leukemias [69,70], mostly in pediatric and infant AML [71,72]. Maseti and colleagues identified eight pediatric patients with *KMT2A* rearrangements, six of whom achieved CR and one of whom achieved a partial response (PR) [62]. During another study, in a cohort of 17 relapsed patients with *KMT2A*, 40% (n = 6) achieved CR/CRi after a median of one cycle of a VTX-including regimen [65]. Preclinical data suggest a significant antiapoptotic dependence and responsiveness to VTX in in vitro models of *KMT2A*-rearranged myeloid and B-cell lymphoblastic leukemia [73,74]. The combination of VTX with novel *KMT2A*-r identified drugs, such as I-BET151, sunitinib, or thioridazine, drastically decreases leukemic cell count, which provides a rationale for targeting the mitochondrial pathway as a strategy to sensitize resistant AML to VTX [74]. Moreover, initial results from a retrospective adult *KMT2A*-rearranged cohort presented a high response rate with VTX and HMA combined therapy [75]. Recent studies concerning adult AML showed increased responses to VTX amongst *NPM1-* [29,76,77,78], *IDH1/2-*, *TET2-*, and relapsed or refractory *RUNX1*-mutated patients, compared to the other cases [77,78]. Therapy for older patients with *NPM1+* AML was associated with CR rates > 85% and an OS of 80% after a median follow-up of 1 year [78]. Somatic mutations of the *NPM1* gene are found in less than 10% of pediatric AML patients, much rarer than in adults [79,80]. Trabal et al. identified six patients with mutation profiles, including *NPM1, IDH1/2*, or *TET2* mutations; three of those children achieved CR/Cri [65]. As of yet, there are no data specific for children regarding markers of VTX sensitivity.

On the contrary, adult AML patients displaying *FLT3, TP53, RAS*, or *PTPN11* mutations, monocytic AML, or AML cases pretreated with HMAs showed decreased receptivity to VTX-based therapies [50,77,81]. During the study by Karol and colleagues, not one of the five patients with *FLT3* alterations responded to treatment [51]. The *FLT3*-tyrosine kinase receptor is vital for normal hematopoietic development. Present in around 30% of cases, somatic activating mutations in this gene are among the most frequent somatic alterations in pediatric AML [82,83]. *FLT3*-internal tandem duplication mutations have been linked to VTX resistance in some experimental models [84]. Gilteritinib, a highly specific inhibitor of *FLT3* mutations, has been certified as a single-agent treatment of relapsed or refractory AML in the United States and Europe [85]. There are emerging clinical trials combining VTX with a tyrosine kinase inhibitor for relapsed and refractory *FLT3+* patients with AML that have demonstrated efficacy [86]. Testing *FLT3* signaling first might be beneficial when identifying a targeted AML population who might respond well to this innovative treatment approach. Further to this, VTX was not efficient in two cases of infant AML with *GLIS* fusions [66], which are correlated with a highly refractory phenotype in pediatric AML subtypes [87,88]. Newly released preclinical data supported VTX resistance in murine models of *CBFA2T3–GLIS2* pediatric acute megakaryoblastic leukemia, but the models displayed sensitivity to navitoclax, a BCL-XL inhibitor, hinting at a potential path for targeting this high-risk infant leukemia [89].

Additional studies are needed to establish if these mutational profiles can guide clinicians on how and when to incorporate VTX into treatment strategies.

## 3. Pediatric ALL

Every year, more progress is made in the treatment of patients with ALL. This is due to the fact that it is the most common childhood cancer, as well as the development of technology and the latest therapy models. The five-year survival rate in patients with de novo ALL has increased in recent years to over 90%, but for patients with relapsed or recurrent ALL, the prognosis is not so favorable, and each subsequent relapse, especially one in which complete remission has not been achieved, brings less hope for recovery [90]. This state of affairs spurs increasing efforts to find new treatments for these patients.

As an imbalance between cell production and cell degradation is typical for cancer, the key in anticancer therapy is to direct pathological cells to the programmed death pathway. VTX, a well-absorbed oral drug, acts by activating the mitochondrial path of apoptosis. Apart from the effect on pathological cells, another research aim was to check whether VTX has a cytotoxic effect on healthy cells. No harmful effects on normal PB cells were noted either in monotherapy [91,92] or in combination with other drugs [91,93].

### 3.1. Comprehensive Treatment with Venetoclax

In a retrospective study by Gibson et al. involving pediatric and AYA patients with relapsed or recurrent hematologic malignancies, including ALL and lymphoblastic lymphoma (LBL), VTX was used as an addition to conventional cytotoxic chemotherapy. Patients received various combinations of cyclophosphamide, vincristine, dexamethasone, doxorubicin, methotrexate, cytarabine, decitabine, nelarabine, pegylated asparaginase, fludarabine, idarubicin, etoposide, gemtuzumab, and rituximab. VTX doses ranged between 100 and 400 mg per day. A total of 61% of patients responded with complete remission. In this study, combination therapy with VTX proved its effectiveness in both first-diagnosed pediatric T-ALL and RR, as well as T-cell LBL [94].

A retrospective observation made by Marinoff et al. showed the efficiency of VTX combined with hypomethylating drugs and chemotherapy in B-ALL patients as well. However, a limitation of that study was that the cohort primarily consisted of relapsed and refractory patients, 70% of whom had received three or more prior lines of therapy. Moreover, the study showed that special attention should be paid to significant side effects such as infections, which were more serious when VTX was combined with conventional chemotherapeutic agents such as vincristine, fludarabine, or cytarabine compared to the combination of VTX with hypomethylating agents such as azacitidine, cytarabine, and decitabine [66]. In addition to infectious side effects, almost 90% of patients presented substantial thrombocytopenia. Most common AEs reported in other studies were severe neutropenia, hyperbilirubinemia, sepsis, aspartate aminotransferase elevation, and disseminated intravascular coagulation [94]. Moreover, as the duration of VTX therapy increased, the thrombocytopenia and neutropenia periods expanded [95]. A similar relationship occurred when increasing the dosage of VTX, despite it not being statistically significant during later observations [95].

The effect of combined therapies with VTX in B-cell ALL seems to exceed the effectiveness of VTX in T-cell ALL. Pullarkat et al., in the evaluation of both pediatric and adult patients with relapsed or recurrent disease after using a combination of VTX, navitoclax, and chemotherapy, noted that the estimated median OS was longer in patients with B-cell ALL than T-cell ALL (9.7 months versus 6.6 months) [96]. It is difficult to determine whether this is solely the effect of VTX due to the fact that, in most clinical trials, VTX is used in combination with other substances to intensify therapy. In another study, after a dosage of VTX (100 mg/kg/for 21 days) in most of the tested pediatric ALL xenografts, an objective response and delay in the development of the disease were achieved. In this case, the treatment contributed to the prolongation of event-free survival (EFS) in B-cell ALL as opposed to T-cell ALL [97]. In a study conducted by Diamanti et al., VTX used in vitro proved to be efficient in most of the samples tested, except for CD34−/CD19− cells, but the in vitro study showed its lack of effectiveness in T-cell ALL. It is important to note that lower BCL-2 expression was observed in T-cell ALL patients, and some authors consider this phenomenon to be a possible cause of lower sensitivity in these patients to VTX [98]. However, the available literature has data on a more successful effect of VTX on T-cell ALL as, in the already-mentioned study by Gibson et al., VTX showed its efficacy in both T-cell ALL and T-cell LBL, where 77% of the patients achieved CR or CRi [94].

It is hard to clearly assess the effectiveness of VTX on specific types of leukemia due to the small number of studied groups of patients, the genetic diversity of patients, and the different treatment regimens used. Table 4 shows data comparing VTX usage in B-cell ALL and T-cell ALL (Table 4).

Brown et al. paid particular attention to VTX increasing the effectiveness of azacitidine in xenografts of neonatal ALL with *KMT2A* rearrangements in a randomized controlled trial. Despite each of these agents having a positive antileukemic effect on its own, it was their combination that proved to be the best option for targeting leukemia stem cells. This highlights the importance of VTX in new therapies, especially in cases of infantile leukemia, which is more aggressive, with event-free survival being much lower than in older children with ALL [99].

Currently, most research focuses on the use of VTX in polytherapy, which will bring more spectacular effects in the fight against cancer, especially in severe cases. Combining VTX, which is a BCL-2-specific inhibitor, with navitoclax, a pan-Bcl-2 inhibitor [98], seems to not only maximize the effectiveness of treatment but also increase clinical tolerance. These conclusions were reached by researchers assessing pediatric and adult patients with relapsed/refractory acute lymphoblastic leukemia or lymphoblastic lymphoma in a multicenter, phase I study (NCT03181126). The study evaluated the safety and preliminary efficacy of VTX with low-dose navitoclax and chemotherapy in 47 patients, 12 of whom were under 18 years of age. The results indicated the validity of combining VTX with low doses of navitoclax in patients with B-cell acute lymphoblastic leukemia, T-cell acute lymphoblastic leukemia, and lymphoblastic lymphoma. The simultaneous inhibition of BCL-2 and BCL-XL led to a reduction in thrombocytopenia and other side effects induced by navitoclax, which ensured treatment effectiveness [96].

There are reports suggesting that BH-3 mimetics are not sufficient in the treatment of childhood leukemias and combining BH-3 mimetics with inhibitors of survival pathways is not a dispensable option [98]. It was proven that the usage of VTX alone, despite a positive response, left leukemic blasts in the liver and spleen and positive minimal residual disease in the bone marrow of mice at the end of therapy [100]. Therefore, a substance that enhances the action of VTX was sought. Researchers identified dinaciclib, which is an inhibitor of cyclin-dependent kinases 1, 2, 5, 9, and 16 showing synergy with VTX, obtaining a 97% inhibition of the growth of leukemic cells in hypodiploid ALL [100]. Such results are promising, but further research in this direction is needed.

Recently, another substance, i.e., a receptor of the tyrosine kinase inhibitor MRX-2843, was discovered, showing synergy with VTX. Combining MRX-2843 with BCL-2 inhibitor showed positive effects in early T-precursor ALL (ETP ALL). This type of leukemia is associated with a high rate of treatment failures. The intensity of action increased in direct proportion to the doses of drugs. Nevertheless, when these substances were used in monotherapy, MRX-2843 showed better effects in ETP ALL [101].

Despite being less effective in monotherapy, VTX should be considered as an agent that intensifies the action of other substances supporting the treatment of hematological cancers. In addition to the dose, different methods of administering VTX with other substances should be considered combinatorial. Richter et al. compared the co-incubation of the new drug MK-2206 with VTX to the administration of one ingredient after the other. In this case, the most satisfactory effect was achieved when VTX was administered second [91]. Such dependencies may occur when other substances are used; therefore, it is important to expand research in this direction.

### 3.2. Venetoclax in Current Clinical Trials

Numerous studies are currently underway assessing the effectiveness of VTX in ALL. Information about ongoing research is summarized in Table 5. Unfortunately, the exact results have not been published yet, but we are hereby looking forward to them, because they may clarify the real benefits of VTX usage in new therapies and influence new treatment options for patients with ALL.

### 3.3. Other Studies on Venetoclax Combination Therapies in ALL

In addition to the above analyses aimed at assessing the validity of VTX usage in contemporary treatment options, there are also case reports on the effectiveness of VTX in combination therapy for pediatric patients with lymphoid malignancies (Table 6) [97,98,99].

## 4. The Mechanisms of Venetoclax Resistance and Future Strategies

There are many known resistance mechanisms to VTX, such as dependencies on other antiapoptotic Bcl-2 family members, *BCL-2* and *BAX* gene mutations, changes within the tumor microenvironment, mitochondrial metabolic reprogramming, and TP53 pathway dysfunction. It is vital to comprehend such mechanisms in order to design reasonable treatment regiments.

It has been proven that persistent VTX exposure leads to the upregulation of different pro-survival Bcl-2 family members, such as BCL-XL, MCL-1, and BCL2-A1 [27,48,76,105,106,107], which have been found to be overexpressed in many hematologic malignancies and other cancers [108,109]. Such proteins are responsible for the sequestration of BIM, which leads to apoptosis inhibition [105,110]. Moreover, VTX treatment resulted in the heightening of MCL-1 protein’s half-life, implying that the association with BIM plays a role in stabilizing Bcl-2 family member proteins [105]. Preclinical studies demonstrated increased levels of MCL-1 and BCL-XL and decreased levels of BCL-2 in VTX-resistant cell lines in comparison with sensitive cell lines both from lymphoid [111] and myeloid [48,112] malignancies. Additionally, after VTX treatment, MCL-1 amplification and overexpression were observed [106]. A study by Niu et al. revealed that incorporating cytarabine or daunorubicin upon VTX treatment resulted in increased DNA damage and a better reduction in MCL-1 levels in AML cell lines than during VTX monotherapy [105]. Moreover, azacitidine correlates with MCL-1 downregulation, which may be the reason for the synergistic outcomes upon combining azacitidine or decitabine and VTX [29,113]. The pharmacological inhibition of BCL-XL, MCL-1, and BCL2-A1 proteins brings back sensitivity to VTX in resistant cells [106,114], which provides a rationale for the further investigation of selective Bcl-2 family member inhibitors as potential therapeutic agents. Toxicity is a restraining factor for BCL-XL inhibitor application due to the reliance on BCL-XL for platelet survival [115]. Despite having a high affinity to BCL-2, BCL-XL, and BCL-W [14], the clinical use of navitoclax (ABT-263) has been restricted by the on-target toxicity of thrombocytopenia [115]. Another study revealed that treatment with the BCL-XL-specific inhibitor WEHI-539 in mouse models led to the lowering of erythrocyte levels and hemoglobin [116]. Among the Bcl-2 family of proteins, MCL-1 protein levels were consistently elevated in virtually all patients with newly diagnosed AML [117]. A study by Teh et al. revealed that the concurrent targeting of BCL2 and MCL1 was associated with long-term survival in AML mouse xenografts, in contrast to targeting a subset of proteins alone [118]. Recently, a selection of MCL-1 inhibitors has been developed, many of which are currently undergoing clinical evaluation: S63845 [119], S64315 [120], AZD5991 [121], AMG-176 [122], and AMG-397 [123]. Furthermore, there are other drugs, such as murine double minute-2 (MDM2), mitogen-activated protein kinase (MEK), or cyclin-dependent kinase 9 (CDK9) inhibitors, that are being investigated for the possible indirect inhibition of MCL-1 [124,125]. Additionally, AML patients’ samples display a differential expression of BCL2-A1 in resistant cells [76,126], which suggests a potential for synergy between BCL-2 and BCL2A1 inhibitors in selective AML subsets [126].

Acquired *BCL-2* mutations, in particular those occurring in the drug binding site, may lead to altered VTX efficacy and to side effects. Several mutations have been identified among VTX-resistant patients with lymphoid malignancies [127,128,129,130]. The G101V mutation is a de novo acquisition mutation, in contrast to pretreatment samples [131], that diminishes the merging of VTX with BCL2 by 180-fold and leads to the outgrowth of blast cells in the presence of the drug in vitro in CLL patients [128]. Nevertheless, among patients with G101V mutations, it is only a cause of part of their resistance to VTX [128]. It is assumed that resistance to VTX depends on multiple, parallel, acquired *BCL2* mutations [132]. Tausch et al. identified a second unreported *BCL2* mutation, D103Y, in one of patients with VTX-refractory CLL [129]. As opposed to the G101 mutation, which prompts BCL-2 to maintain the ability to bind to the BH3 motifs in the regulatory protein, the D103 mutation is part of the BH3 binding pocket of BCL2 [128,129]. With the previous data of acquired resistance to VTX due to Phe104 mutations [133], the Phe104Ile mutation was detected in a patient with relapsed/refractory follicular lymphoma (FL) treated with BCL-2 inhibitor, which lessened the binding of VTX to BCL-2 by approximately 40-fold [127].

The fairly frequently reoccurring mechanism of treatment resistance in lymphoid malignancies, especially CLL, which is clinically sensitive to VTX monotherapy [33,34,35], is the previously mentioned *BCL-2* mutation. On the contrary, in myeloid malignancies, where VTX combination therapies are much more applicable, more wide-ranging patterns of resistance have been discovered. Acquired *BAX* mutations that either disrupted protein expression or its proapoptotic function led to adaptive resistance to BCL-2 inhibitors in AML patients who relapsed after VTX-combined therapies [134]. Such genetic changes were rarely detectable in patients who relapsed upon conventional chemotherapy [134]. VTX is able to induce apoptosis in cells with the normal *BAK* gene, on the contrary to cells that lack *BAX*, suggesting a significance of the *BAX* gene in VTX-induced apoptosis [135,136]. A study by Moujalled et al. discovered resistance to both BCL-2 and MCL-1 (S63845) inhibitors amongst cells with *BAX* mutations [134]. Another study similarly presented resistance to BCL-2 and the BCL-2/BCL-XL (AZD-4320) inhibitor, but sensitivity to the MCL-1 inhibitor (AZD-5991) [135]. Moreover, several *BAX* mutations have been identified amongst VTX-resistant CLL patients [137,138].

There are few VTX-resistance mechanisms connected to the tumor microenvironment. The activation of the B-cell receptor (BCR) by a BCL-2 inhibitor [139], as well as initializing the extracellular-signal-regulated kinase (ERK) pathway via the activation of extracellular receptors by ABT-737 [140], leads to the upregulation of MCL-1 protein. Moreover, the CD40L/CD40-mediated stimulation of CLL blasts by activated T-cells in the lymph node microenvironment prompts increased antiapoptotic protein expression (MCL1, BCL-XL, and BFL-1) [106,141,142]. In contrast to MCL-1, the knockdown of BCL-XL greatly altered the sensitivity of leukemic cells to VTX after CD40 stimulation [106]. Anti-CD20 monoclonal antibodies such as GA101 [143] or rituximab [144], the c-Abelson tyrosine kinase (c-Abl) inhibitors imatinib or dasatynib [144], or Bruton’s tyrosine kinase (BTK) inhibitors [145] induce non-apoptotic cell death in CD40-stimulated CLL cells and, in turn, restore VTX sensitivity.

A study by Kuusanmäki et al. showed a connection between VTX sensitivity and the degree of AML cell differentiation. Blasts proved to be highly sensitive to BCL-2 inhibition, whereas monocytes and granulocytes showed resistance. Cells with the French–American–British (FAB) subtype M4/5 AML were characterized by the highest resistance to VTX, which can be associated with the lowest expression of BCL-2 and the strongest expression of MCL-1 and BCL2-A1. An inverse relationship was observed among FAB-M0 cells. Additionally, researchers found that resistant blasts were sensitive to either MEK and/or Janus kinase (JAK) inhibitors. Therefore, a way to counteract VTX resistance may be the additional use of a MEK inhibitor or a JAK inhibitor [146]. Similar conclusions about the connection between the increase in reluctance with the degree of cell differentiation were reached by Pei et al. Moreover, their univariate analysis showed that gender and the presence of RAS pathway mutations were also associated with VTX + AZA resistance. It was also noted that leukemic cells at different developmental stages consist of separate mechanisms to mediate energy metabolism, and in monocytic AML MCL-1, reliance shows its importance. Therefore, AML cells can switch from BCL-2 to MCL-1 dependence to drive energy metabolism as cells obtain a higher developmental state. In summary, the authors point out that MCL-1 inhibition may be one of the strategies to counteract resistance to the used treatment and the subject of further research [147].

Another mechanism of VTX resistance is mitochondrial metabolic reprogramming. The *TP53* mutation in leukemic stem cells disturbed mitochondrial homeostasis by impairing the effector function of BAX/BAK and hindered BCL-2 expression, which decreased the target of VTX [135]. An abnormal overexpression of components of the AMP-activated protein kinase (AMPK)/protein kinase A (PKA) pathway was observed in the CLL cell line OCI-Ly1 with an amplification of 1q23, which resulted in resistance to VTX [111]. After using a genome-wide CRISPR knockout screen to find genes that, upon inactivation, restored sensitivity to VTX in an AML cell line with acquired resistance, Sharon et al. found that gene inactivation involving mitochondrial translation sensitized resistant AML cells. The combined use of drugs which target the mitochondrial respiratory chain can further enhance the anti-AML effect of VTX [148].

Genomic instability is a different mechanism of possible VTX resistance. *TP53* biallelic mutations were commonly described in patients with both primary and adaptive resistance to VTX-based therapies [149]. Moreover, the acquisition of new *TP53* mutations during the course of AML therapy has been reported [150]. Preclinical studies demonstrated resistance to both BCL-2 and MCL-1 inhibitors in *TP53*-mutated cells and xenograft models [151], which could be restored by the concurrent inhibition of both Bcl-2 family members [152]. Such findings suggest that combining VTX with an MCL-1 inhibitor could be an alternative to bypass resistance associated with *TP53* mutation. There are new drugs being investigated, such as eprenetapopt (APR-246), that reactivate the mutant p53 protein and target cellular redox balance, in order to bring back apoptosis in *TP53*-mutated cancer cells. Combined results of two parallel phase II trials combining APR-246 with AZA showed the combination to be well tolerated, with high response rates in m *TP53* MDS/AML [153]. There is an ongoing phase I clinical trial (NCT04214860) testing the combination of APR-246, AZA, and VTX.

As mentioned previously, *FLT3-ITD* or *PTPN11* mutations that result in the activation of intracellular signaling pathways correlate with VTX resistance [50,77,81,84]. Moreover, such mutations were acquired by a subset of AML patients during disease relapse, which indicated that these mutations are linked to secondary VTX resistance [84]. Studies have shown that *FLT3-ITD* or *PTPN11* mutations cause an elevation of BCL-XL and MCL-1 protein levels [154,155,156], which may explain the resistance mechanism. The MCL-1 protein escalation, as well as BAD and BIM suppression, is a result of the activation of different downstream signaling events involving the phosphoinositide 3-kinase (PI3K)/protein kinase B (AKT)/mechanistic target of rapamycin (mTOR) and MEK/ERK pathways, and signal transducers and activators of transcription 5 (STAT5), leading to cytokine-independent cell survival and proliferation [157]. A study by Yoshimoto et al. demonstrated a reduction in MCL-1 protein levels in *FLT3-ITD* MV4-11 AML cells after incubation with STAT5 small interfering RNA (siRNA), which highlights the importance of FLT3-STAT5 for MCL-1 expression [156]. The suppression of FLT3-STAT5 with the multikinase inhibitor olverembatinib (HQP1351) caused *FLT3-ITD* mutant AML cell growth reduction. A combination of the BCL-2 inhibitor APG-2575 with HQP1351 resulted in the downregulation of MCL-1, which led to the strengthening of APG-2575-induced apoptosis [158]. Moreover, the addition of the small-molecule FLT3 inhibitor quizartinib upon VTX treatment prompted more durable responses in mice *FLT3-ITD+* mutant xenograft MV-4–11 cells than either agent alone [84]. Combining FLT3 inhibitors with VTX could be a useful strategy to overcome BCL-2 inhibitor resistance between *FLT3*-mutated AML patients and to prevent the appearance of *FLT3*-mutated subclones in patients with no detectable *FLT3* mutation.

There are no data specific for pediatric populations regarding the mechanism of VTX resistance and markers of VTX response. Therefore, populations of patients with unsatisfying treatment response or disease progression upon BCL-2 inhibition should be further studied in both preclinical models and in clinical trials.

## 5. Conclusions

In summary, targeting the apoptosis pathway by inhibiting Bcl-2 family proteins with VTX in combination with established drugs in the treatment of leukemia is a highly promising strategy to improve survival and reduce treatment-related toxicities for a specific subgroup of AML or ALL pediatric patients. Based on the latest data, such regimens can be used both as an emergency therapy in the case of treatment-resistant leukemias and, most importantly, as a bridge therapy if it is necessary to perform an allogeneic hematopoietic stem cell transplant. Despite infectious complications being the most common adverse events, treatment can be well-tolerated. The overall response rate is comparable to that seen in heavily pretreated adult patients who received similar VTX combination therapies. It is crucial to determine markers of VTX response, as there are no data specific for pediatric populations, whereas genetic variants/lesions associated with VTX sensitivity or its reduction from adult studies are not frequent or applicable in children. Further studies are needed to identify the various mechanisms of VTX resistance and methods of overcoming them. International cooperation in the form of multicenter studies is necessary to gather larger, homogenous research groups to identify an optimal VTX-based regimen for the pediatric population and long-term effects on patients’ outcomes.

## Figures and Tables

**Table 2:** Venetoclax-based AML therapies in pediatric population in ongoing registered clinical trials.

NCT	Study	Intervention/ Treatment	Phase	Condition/ Disease	Study Start	Estimated/ Actual Enrollment	Study Group
04161885	A Study Evaluating Safety and Efficacy of Venetoclax in Combination With Azacitidine Versus Standard of Care After Allogeneic Stem Cell Transplantation in Participants With Acute Myeloid Leukemia	VTX; AZA	III	AML	26 February 2020	424 participants	12 years and older
03941964	A Study of the Effectiveness of Venetoclax in Combination With Azacitidine or Decitabine in an Outpatient Setting in Patients With Acute Myeloid Leukemia Ineligible for Intensive Chemotherapy	VTX; AZA; DEC	III	AML	15 August 2019	60 participants	12 years and older
02250937	Venetoclax and Sequential Busulfan, Cladribine, and Fludarabine Phosphate Before Donor Stem Cell Transplant in Treating Patients With Acute Myelogenous Leukemia or Myelodysplastic Syndrome	VTX; BUS; CLAD; FLU	II	AML and myelodysplastic syndrome	27 October 2014	116 participants	2 years to 70 years
04029688	A Study Evaluating the Safety, Tolerability, Pharmacokinetics and Preliminary Activity of Idasanutlin in Combination With Either Chemotherapy or Venetoclax in the Treatment of Pediatric and Young Adult Participants With Relapsed/Refractory Acute Leukemias or Solid Tumors	VTX; CYT; FLU; TOP; IDA; CYC	I, II	AML, ALL, neuroblastoma, and solid tumors	27 January 2020	183 participants	0 years to 30 years
04000698	Personalized Targeted Preparative Regimen Before T-depleted Allogeneic HSCT in Children With Chemoresistent Acute Leukemias	Preparative chemotherapy before allogeneic HSCT	III	Refractory AML and refractory ALL	15 October 2019	25 participants	0 years to 25 years
03844048	An Extension Study of Venetoclax for Subjects Who Have Completed a Prior Venetoclax Clinical Trial	VTX	III	CLL, AML, MM, non-Hodgkin’s lymphoma, ALL, and cancer	6 September 2019	550 participants	Children, adults, and older adults
03826992	Venetoclax Combined With Vyxeos for Participants With Relapsed or Refractory Acute Leukemia	VTX; VYX	I	Leukemia	27 December 2018	21 participants	1 year to 39 years

AZA—azacitidine, DEC—decitabine; BUS—busulfan; CLAD—cladribine; FLU—fludarabine; CYT—cytarabine; TOP—topotecan; IDA—idasanutlin; CYC—cyclophosphamide; VYX—Vyxeos.

**Table 3:** Case series reporting results on the use of venetoclax in the treatment of myeloid malignancies in children.

Age/Sex of Patients	Disease	Intervention/ Treatment	Therapy Effect	Adverse Events	References
12 years/ Male	Relapsed pediatric mixed-phenotype acute leukemia	VTX + AZA	Unfulfillment of the second HSCT criterion; blast escalation; and death after 8 months post-relapse	Febrile neutropenia and lung aspergillosis	Gonzales et al. [53]
17 months/ female	*CBFA2T3/GLIS2* relapsed acute megakaryoblastic leukemia	VTX + AZA	MRD negative remission after one cycle; sustained through six cycles	Neutropenia	Mishra et al. [54]
5 years/ male	*NUP98-NSD1+*/*FLT3-ITD+* acute myeloid leukemia	VDAH; VDA	Proceeded to an allogeneic HSCT; in remission 301 days post-transplantation	No information	Wen et al. [55]
3 years/ male	Refractory *NUP98-NSD1* fusion acute myeloid leukemia	DCAG + VTX	Proceeded to an allogeneic HSCT; in remission 6 months post-transplantation	No information	Xu et al. [56]
16 years/ male	Relapsed acute myeloid leukemia in Shwachman–Diamond syndrome arising from MDS	VTX + AZA	Partial bone marrow response; AML progression; and death from multi-organ failure	Diarrhea, peripheral edema, urticarial rash gingivostomatitis, and sepsis	Naviglio et al. [57]
3 years/ male	Acute myeloid leukemia with myelodysplasia-related changes	VTX + AZA	Proceeded to an allogeneic HSCT; in remission 18 months post-transplantation	Myelosuppression	Wen et al. [58]
5 years/ female	*NPM1*-mutated donor-derived MDS/AML in a patient with Fanconi anemia	VTX + AZA	Proceeded to an HSCT; in remission 1.5 years post-transplantation	Neutropenia, subdural hematoma, and pulmonary infection	Ma et al. [59]

AZA—azacitidine; VDAH—venetoclax, dasatinib, cytarabine, and homoharringtonine; VDA—venetoclax, dasatinib, and azacitidine; DCAG—decitabine, aclacinomycin, cytosine arabinoside, and granulocyte colony-stimulating factor.

**Table 4:** Patient disease characteristics, concurrent therapy, response, and toxicity [66,94].

Patient Number	Diagnosis	Age/Sex	Treatment Combined with VTX	Best Response	Adverse Events
1	Relapsed B-cell ALL	20/F	VCR/PEG/DEX	CR	Paronychia, thrombocytopenia, anemia, and neutropenia
2	Relapsed B-cell ALL	27/M	FLAG	PD	Sepsis, thrombocytopenia, anemia, and neutropenia
3	Relapsed B-cell ALL	15/F	VCR/PEG/DEX	CR	Sepsis, thrombocytopenia, anemia, and neutropenia
4	B-cell ALL	21/F	CVD	CRi	Febrile neutropenia and thrombocytopenia
5	B-cell ALL	18/M	CVD	NR	Thrombocytopenia and neutropenia
6	B-cell ALL	11/M	CVD	NR	Myelosuppression and hyperbilirubinemia
7	B-cell ALL	20/F	HyperCVAD and RUX	NR	Thrombocytopenia
8	B-cell ALL	6/F	CVD	NR	Thrombocytopenia, sepsis, and hyperbilirubinemia
9	T-cell LBL	12/M	HyperCVAD and DEC	NR	Thrombocytopenia
10	T-cell LBL	20/M	DEC	CR	Febrile neutropenia, thrombocytopenia, and coagulopathy
11	T-cell LBL	20/M	HyperCVAD and NEL	CR	Sepsis and pancreatitis
12	T-cell LBL	20/F	HyperCVAD, NEL, and PEG	CR	Thrombocytopenia and neutropenia
13	T-cell LBL	21/M	HyperCVAD, NEL, and FLAG	CR	Thrombocytopenia
14	T-cell LBL	21/F	CYT, IDA, and PEG	CR	Febrile neutropenia, sepsis, and myelosuppression
15	T-cell ALL	21/M	NEL, ETO, CYC, and DEC	NR	Pneumonia, sepsis, thrombocytopenia, and hyperbilirubinemia
16	T-cell ALL	19/M	HyperCVAD	CR	Thrombocytopenia, sepsis, and hyperbilirubinemia
17	T-cell ALL	17/M	HyperCVAD	CRi	Febrile neutropenia and thrombocytopenia
18	ETP T-cell ALL	19/M	FLAG, CYT, GEM, and MTX	CR	Thrombocytopenia and neutropenia
19	T-cell ALL	18/M	HyperCVAD	NR	Thrombocytopenia and neutropenia
20	T-cell ALL	21/M	HyperCVAD, NEL, and PEG	CR	Febrile neutropenia and thrombocytopenia
21	T-cell ALL	22/M	NEL, PEG, and GEM	CR	None

CR—complete remission; PD—progressive disease; NR—no response; CRi—complete remission without blood count recovery; VCR—vincristine; PEG—pegaspargase; DEX—dexamethasone; FLAG—fludarabine; CVD—cyclophosphamide, vincristine, and dexamethasone; HyperCVAD—hyper-fractioned cyclophosphamide, vincristine, dexamethasone, doxorubicin, methotrexate, and cytarabine; RUX—ruxolitinib; DEC—decitabine; NEL—nelarabine; CYT—cytarabine; IDA—idarubicin; ETO—etoposide; CYC—cyclophosphamide; GEM—gemtuzumab; MTX—methotrexate.

**Table 5:** Venetoclax-based ALL therapies in pediatric population in clinical trials.

NCT	Study	Intervention/ Treatment	Phase	Condition/ Disease	Study Start	Estimated/ Actual Enrollment	Study Group
03236857	A Study of the Safety and Pharmacokinetics of Venetoclax in Pediatric and Young Adult Patients With Relapsed or Refractory Malignancies	VTX; chemotherapy	I	AML, ALL, non-Hodgkin’s lymphoma, and neuroblastoma	8 November 2017	143 participants	0 years to 25 years
04029688	A Study Evaluating the Safety, Tolerability, Pharmacokinetics and Preliminary Activity of Idasanutlin in Combination With Either Chemotherapy or Venetoclax in Treatment of Pediatric and Young Adult Participants With Relapsed/Refractory Acute Leukemias or Solid Tumors	IDA; VTX; and chemotherapy	I, II	AML, ALL, neuroblastoma, and solid tumors	27 January 2020	183 participants	0 years to 30 years
05386576	Venetoclax in Combination With Asparaginase-Containing Pediatric-Inspired Chemotherapy in Adult Patients With Newly Diagnosed Acute Lymphoblastic Leukemia	VTX	I	ALL	16 June 2022	12 participants	18 years to 60 years
03181126	A Phase 1 Dose Escalation, Open-Label Study of Venetoclax in Combination With Navitoclax and Chemotherapy in Subjects With Relapsed/Refractory Acute Lymphoblastic Leukemia or Relapsed/Refractory Lymphoblastic Lymphoma	VTX; NAV; and chemotherapy	I	ALL and LL	27 November 2017	69 participants	4 years and older
05660473	Pediatric-inspired Regimen Combined With Venetoclax for Adolescent and Adult Patients With de Novo Philadelphia Chromosome-Negative Acute Lymphoblastic Leukemia	VTX; chemotherapy	II	Precursor cell lymphoblastic leukemia-lymphoma	31 October 2022	100 participants	14 years to 60 years
05740449	International Proof of Concept Therapeutic Stratification Trial of Molecular Anomalies in Relapsed or Refractory HEMatological Malignancies in Children, Subprotocol A: Decitabine/Venetoclax and Navitoclax in Pediatric Patients With Relapsed or Refractory Hematological Malignancies	DEC; VTX; and NAV	I, II	Relapsed/refractory ALL	1 October 2023	26 participants	1 year to 21 years
05745714	International Proof of Concept Therapeutic Stratification Trial of Molecular Anomalies in Relapsed or Refractory HEMatological Malignancies in Children, Subprotocol C Ruxolitinib + Venetoclax + Dexamethasone + Cyclophosphamide and Cytarabine in Pediatric Patients With Relapsed or Refractory Hematological Malignancies	RUX; VTX; DEXA; CP; and Ara C	I, II	Relapsed/refractory ALL	1 October 2023	26 participants	1 year to 21 years
05751044	International Proof of Concept Therapeutic Stratification Trial of Molecular Anomalies in Relapsed or Refractory HEMatological Malignancies in Children, Sub-protocol B Dasatinib + Venetoclax + Dexamethasone + Cyclophosphamide and Cytarabine in Pediatric Patients With Relapsed or Refractory Hematological Malignancies	DAS; VTX; DEXA; CP; and Ara C	I, II	Relapsed/refractory ALL	1 October 2023	26 participants	1 year to 21 years
05157971	Study Combining Venetoclax With a Pediatric-Inspired Regimen for Newly Diagnosed Adults With B Cell Ph-Like Acute Lymphoblastic Leukemia	VTX; Pred; and chemotherapy	I	BALL and Ph-like ALL	17 March 2022	Six participants	18 years to 54 years

VTX—venetoclax, IDA—idasanutlin, NAV—navitoclax, DEC—decitabine, RUX—ruxolitinib, CP—cyclophosphamide, DEXA—dexamethasone, Ara C—cytarabine, DAS—dasatinib, Pred—prednisone.

**Table 6:** Case series reporting results on the use of venetoclax in the treatment of lymphoid malignancies in children.

Age/Sex of Patients	Disease	Intervention/ Treatment	Therapy Effect	Adverse Events	References
15 years/ male	Chronic myelomonocytic leukemia with germline *GATA2* mutation	VTX + DEC	Proceeded to a myeloablative, haploidentical peripheral blood stem cell (PBSC) transplantation; in remission 1 year post-transplantation	Nausea, neutropenia, anemia, and thrombocytopenia	Molina et al. [102]
11 years/ male	Relapsed blastic plasmacytoid dendritic cell neoplasm	CVAD + VTX	Proceeded to an allogeneic HSCT; in remission 200 days post-transplantation	Pneumonia, febrile neutropenia, and bacteremia	Abla et al. [103]
16 years/ male	t(17;19) acute lymphoblastic leukemia	VTX + NAV	Proceeded to an allogeneic HSCT; septic shock at day + 10; and death by multi-organ failure	Neutropenia, thrombocytopenia, and diarrhea	Gottardi et al. [104]

DEC—decitabine; CVAD—cyclophosphamide, vincristine, doxorubicin, dexamethasone alternating with methotrexate, and cytarabine; NAV—navitoclax.

## Data Availability

Not applicable.
